# Quantification of dynamic contrast-enhanced ultrasound (CEUS) in non-cystic breast lesions using external perfusion software

**DOI:** 10.1038/s41598-021-96137-6

**Published:** 2021-09-03

**Authors:** Ernst Michael Jung, Friedrich Jung, Christian Stroszczynski, Isabel Wiesinger

**Affiliations:** 1 Institute of Radiology, Interdisciplinary Department for Ultrasound, University Medical Center, Regensburg, Franz-Josef-Strauß-Allee 11 , 93053 Regensburg, Germany; 2 Institute of Biotechnology, Molecular Cell Biology, Brandenburg University of Technology, Universitätsplatz 1, 01968 Senftenberg, Germany

**Keywords:** Oncology, Cancer imaging

## Abstract

The aim of this present clinical pilot study is the display of typical perfusion results in patients with solid, non-cystic breast lesions. The lesions were characterized using contrast enhanced ultrasound (CEUS) with (i) time intensity curve analyses (TIC) and (ii) parametric color maps. The 24 asymptomatic patients included were genetically tested for having an elevated risk for breast cancer. At a center of early detection of familial ovary and breast cancer, those patients received annual MRI and grey-scale ultrasound. If lesions remained unclear or appeared even suspicious, those patients also received CEUS. CEUS was performed after intravenous application of sulfur hexafluoride microbubbles. Digital DICOM cine loops were continuously stored for one minute in PACS (picture archiving and communication system). Perfusion images and TIC analyses were calculated off-line with external perfusion software (VueBox). The lesion diameter ranged between 7 and 15 mm (mean 11 ± 3 mm). Five hypoechoic irregular lesions were scars, 6 lesions were benign and 12 lesions were highly suspicious for breast cancer with irregular enhancement at the margins and a partial wash out. In those 12 cases, histopathology confirmed breast cancer. All the suspicious lesions were correctly identified visually. For the perfusion analysis only Peak Enhancement (PE) and Area Under the Curve (AUC) added more information for correctly identifying the lesions. Typical for benign lesions is a prolonged contrast agent enhancement with lower PE and prolonged wash out, while scars are characterized typically by a reduced enhancement in the center. No differences (p = 0.428) were found in PE in the center of benign lesions (64.2 ± 28.9 dB), malignant lesions (88.1 ± 93.6 dB) and a scar (40.0 ± 17.0 dB). No significant differences (p = 0.174) were found for PE values at the margin of benign lesions (96.4 ± 144.9 dB), malignant lesions (54.3 ± 86.2 dB) or scar tissue (203.8 ± 218.9 dB). Significant differences (p < 0.001) were found in PE of the surrounding tissue when comparing benign lesions (33.6 ± 25.2 dB) to malignant lesions (15.7 ± 36.3 dB) and scars (277.2 ± 199.9 dB). No differences (p = 0.821) were found in AUC in the center of benign lesions (391.3 ± 213.7), malignant lesions (314.7 ± 643.9) and a scar (213.1 ± 124.5). No differences (p = 0.601) were found in AUC values of the margin of benign lesions (313.3 ± 372.8), malignant lesions (272.6 ± 566.4) or scar tissue (695.0 ± 360.6). Significant differences (p < 0.01) were found in AUC of the surrounding tissue for benign lesions (151.7 ± 127.8), malignant lesions (177.9 ± 1345.6) and scars (1091 ± 693.3). There were no differences in perfusion evaluation for mean transit time (mTT), rise time (RT) and time to peak (TTP) when comparing the center to the margins and the surrounding tissue. The CEUS perfusion parameters PE and AUC allow a very good assessment of the risk of malignant breast lesions and thus a downgrading of BI-RADS 4 lesions. The use of the external perfusion software (VueBox, Bracco, Milan, Italy) did not lead to any further improvement in the diagnosis of suspicious breast lesions and does appears not to have any additional diagnostic value in breast lesions.

## Introduction

Breast cancer is one of the most frequently diagnosed cancers worldwide, following lung cancer. If detected at an early stage, the chances of curative treatment and preservation of good quality of live are high. Usually, the first screening step is done by mammography. Especially in dense breast tissue (ACR IV/d) the sensitivity of mammography is low and may result in false negative results when compared to less dense breast tissue^1,2^. Furthermore, lesions may remain unclear. The second step in the diagnostic work-up is mostly conventional ultrasound. In younger women it may even be the first step^3^.

Up to now as for contrast-enhanced imaging methods, MRI is still restricted to preselected indications such as in a high-risk familial breast cancer setting^4^ in combination with high-resolution conventional grey-scale ultrasound.

According to the guidelines by the European Federation of Societies for Ultrasound in Medicine and Biology (EFSUMB) with the update in 2017, contrast-enhanced ultrasound (CEUS) for the characterization of breast lesions is an active field of research but is not yet recommended for clinical use. Neither is there any recommendation of intradermal injection of contrast media to identify the sentinel lymph node, although it is currently an active field of research^5^.

Various recent international studies describe CEUS as highly sensitive for the characterization of benign and malignant breast lesions for the evaluation of tumor response during and after neoadjuvant chemotherapy, for the differentiation between post-operative scars and recurrence and for the evaluation of lymph nodes^6–11^.

CEUS enables a dynamic evaluation of micro-vascularization down to the capillaries when using high resolution probes^12^. In recent literature, many applications and techniques are described for amplification of the echo signal in ultrasound diagnostics for breast tumors. Recently contrast harmonic imaging (CHI) is more frequently used together with second generation contrast agent (SonoVue, Bracco, Milan, Italy). The first-generation contrast media was too fragile when used with high frequencies. As in all CEUS examinations, a low mechanical index (MI < 0.16) is used, consequently the echo power is amplified due to oscillation of the bubbles. Hence, the neoangiogenesis in and around the tumor can be displayed as a criterion for the proliferation rate of the tumor. Ergo, CEUS can be utilized to rate non-cystic lesions that cannot be assessed in conventional (B-Mode) ultrasound alone. Tumor-specific contrast-media has not yet been approved although it is in trials^13^.

However, so far, the use of CEUS in breast imaging is still the exception, since US-BI-RADS only takes conventional B-Mode images into consideration. Still, CEUS in breast imaging can be seen as helpful if taking all the risk factors of gadolinium into account, when MRI is performed in high-risk situation, or in dense breast tissue (ACR c/d) for multimodal imaging.

A further step in CEUS diagnostics is the independent reading of cine loops with external perfusion software (e.g. VueBox, Bracco, Milan, Italy). The aim of this present clinical pilot study is the display of typical perfusion results, characterization with time intensity curve analyses (TIC), evaluation of parametric color maps and comparison with CEUS literature in cases of solid, non-cystic breast lesions.

## Materials and methods

Before all contrast-enhanced examinations were written informed consent from the patients was obtained. The study was waived by the ethical board at the University Medical Center Regensburg (20-2122-104). All the images were stored in PACS for independent reading. This study was performed in accordance with the Declaration of Helsinki.

All the patients in this study were genetically tested for having an elevated risk for breast cancer. The patients were classified as high lifetime risk for having breast cancer if a BRCA 1 or 2, TP53, or PALB2 mutation was found. A moderate risk was calculated for patients with RAD51D or RAD51C, CHEK2, NBN, ATM or CDH1 mutation. Furthermore, there was a group of elevated lifetime risk for having breast cancer without any of the above-mentioned mutations. At a center of early detection of familial ovary and breast cancer, those patients received a ceMRI annually, followed by conventional grey scale ultrasound (every 6–12 months). If lesions remained unclear or appeared even suspicious, those patients also received CEUS. Mammography was also performed with preselected indications but will not be dealt with in this study. The patients were examined between January 2019 and December 2020. They all had lesions that remained unclear after MRI and conventional ultrasound. They were all visible on ultrasound but could not be classified with conventional B-Mode Ultrasound.

For MRI a 1.5 T scanner was used (Avanto, Siemens, Erlangen, Germany). The patients were placed in prone position and contrast media (6–10 ml Gadovist 1.0 mmol/ml, Bayer, Leverkusen, Germany) was applied according to their body weight. The protocol contained diffusion weighted imaging (DWI), ADC maps, T2 STIR, as well as native and dynamic contrast-enhanced T1 flash sequences with subtraction imaging.

Grey scale ultrasound was performed using a high-end machine (LOGIQ E9, GE Healthcare, Solingen, Germany) with a high-resolution linear transducer (ML 6–15 MHz for B-Mode and L 6–9 MHz for CEUS). The patients were placed in supine position for ultrasound with arms placed above or under the head. First the axilla was scanned and pictures of lymph nodes were documented. Then the whole breast was scanned, and at least one picture was documented for each quadrant. If lesions were seen, except for those that were clearly identified as bland cysts, they were documented in three planes and Doppler ultrasound was performed to assess the vascularization. If those lesions remained unclear after MRI and conventional greyscale ultrasound, CEUS was performed after i.v. injection of 2.4 ml sulfur hexafluoride microbubbles (SonoVue/Lumason in the US, Bracco, Milan, Italy) via a cubital vein, followed by a 10 ml saline flush. The CEUS examinations were performed by one experienced examiner (> 3000 scans/year, > 20 years of experience). The standard presets for CEUS with contrast harmonic imaging (CHI), tissue harmonic imaging (THI), pulse inversion harmonic imaging (PIHI) and a low mechanical index (MI < 0.16) were used. Digital DICOM loops were continuously stored for one minute in picture archiving and communication system (PACS). For the arterial phase short loops were continuously stored for one minute and afterwards single images were stored up to the late phase (3 min after contrast injection).

For a detailed perfusion analysis those loops were uploaded and opened in VueBox (Bracco, Milan, Italy) on a separate computer for independent reading. The reading was independently performed by two experienced radiologists in consensus.

**Figure 1:**
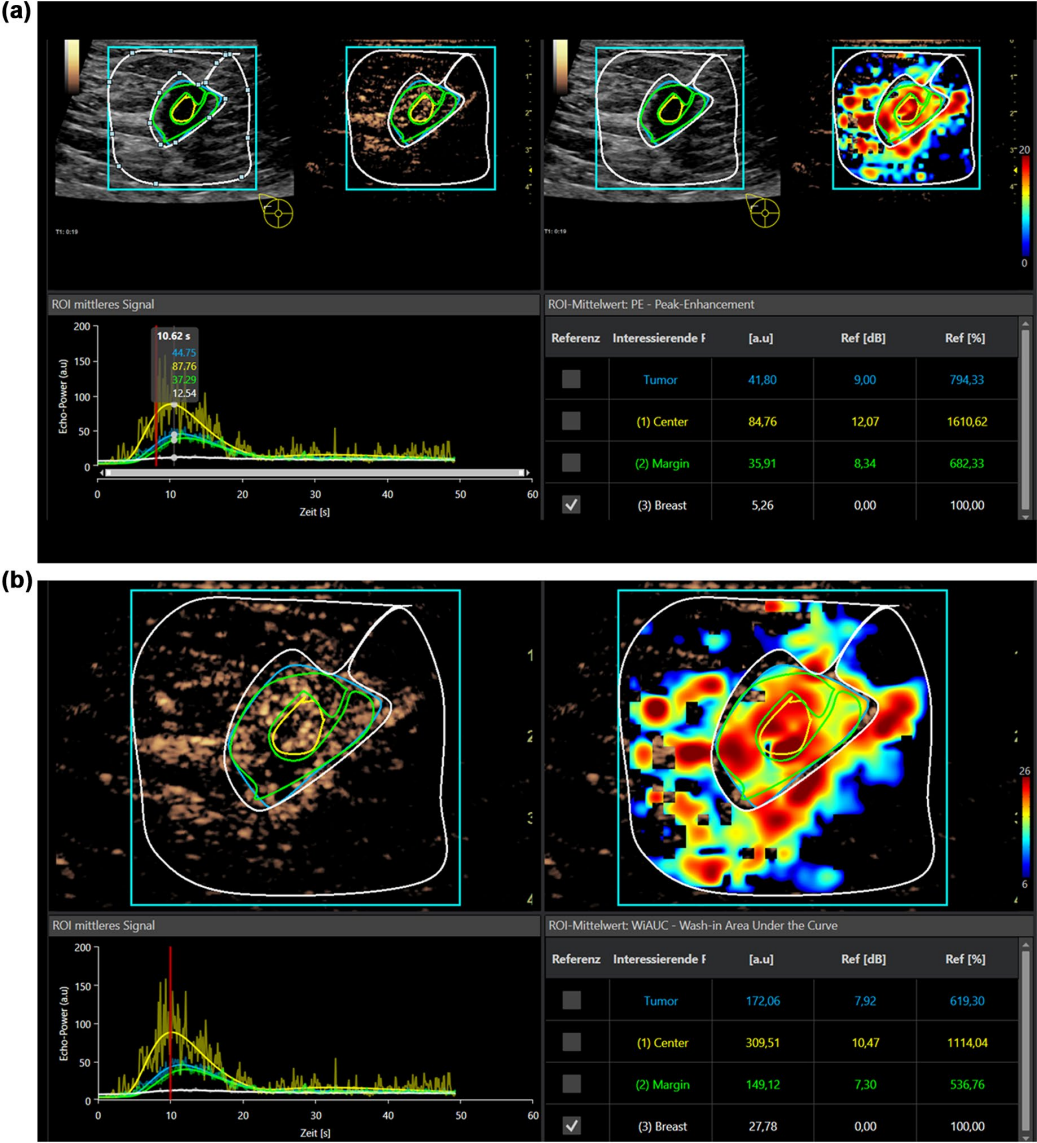

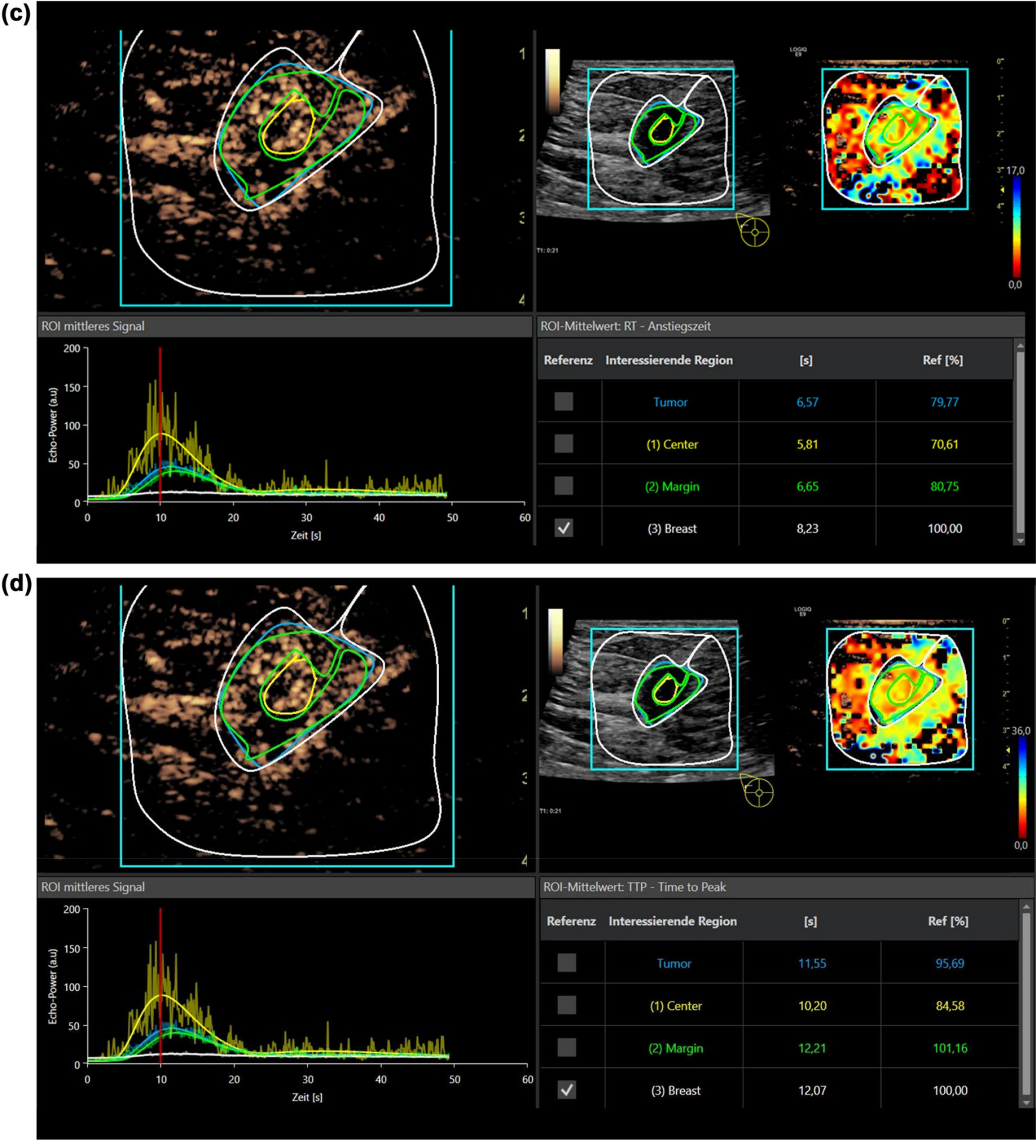
(**a**) Evaluation of dynamic CEUS micro-vascularization of a malignant breast lesion after i.v. application of 2.4 ml ultrasound contrast media using external perfusion software VueBox. The VueBox screen is divided into four sections. Up left, the original CEUS image with the manually drawn Regions of Interest (ROI). Up right, the perfusion imaging with false colors showing hyperperfusion in red shades. Down left, the Time Intensity Curve analysis (TIC) of the Regions of Interest and down right the numeric values of the regions. The healthy breast tissue is taken as reference. Each perfusion parameter is calculated in an extra step. Case of a small malignant breast tumor. Contrast enhanced evaluation up to 60 s for PE. In parametric imaging irregular red and yellow false colors for tumor micro-vascularization, blue for the surrounding tissue. High and fast wash in and rapid wash out in time intensity curve analysis (TIC) as criteria for malignancy. (**b**) Same case of a malignant breast lesion with evaluation of wash in area under the curve (AUC). (**c**) Same case of a malignant breast lesion with evaluation of Rise Time (RT). Less differences are seen in parametric imaging compared to the surrounding tissue. (**d**) Same case of a malignant breast lesion with evaluation of Time to Peak (TTP), with hardly any differences in parametric imaging compared to the surrounding tissue.

**Figure 2:**
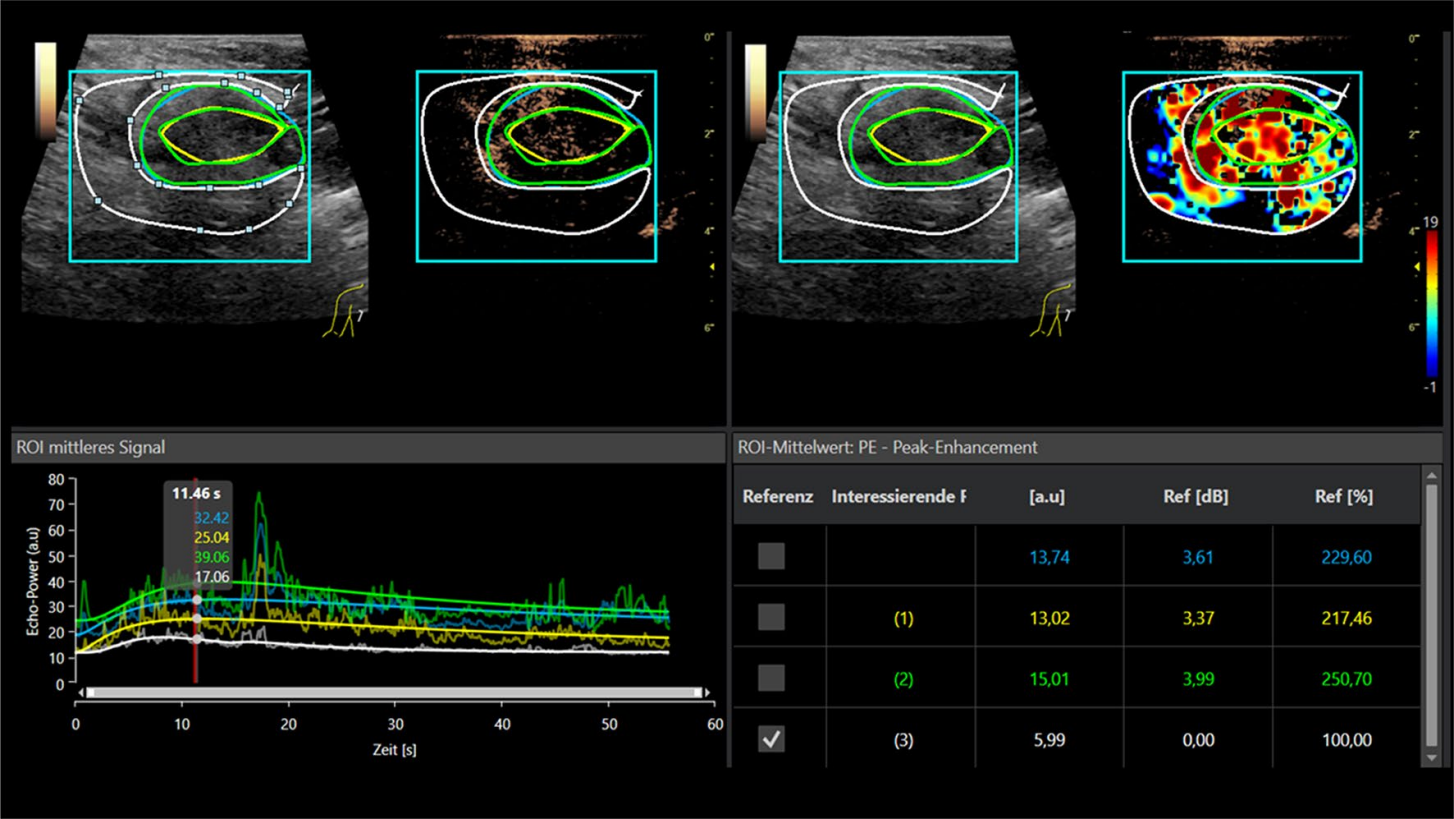
CEUS Perfusion evaluation of a small benign breast tumor, histo-pathologically proven fibroadenoma. Contrast enhanced perfusion evaluation up to 60 s for measurement of the PE. In parametric imaging irregular red and yellow shades for tumor micro-vascularization. There is hardly any difference visible when compared to the surrounding tissue (also in yellow shades). Slower wash in rate, lower peak and prolonged wash out in time intensity curve analysis (TIC) as criteria for a benign breast tumor.

VueBox is a color-coded off-line general-purpose perfusion software for dynamic CEUS examinations with integrated motion correction. Regions of interest (ROI) have to be defined and are manually placed in the center of the suspicious area, at the margins and in the surrounding tissue (Figs. 1a–d, 2). Afterwards, the software automatically calculates PE, wash in rate, TTP, MTT, RT, and AUC. A ROI is also placed in the surrounding breast tissue as reference for all parameters. The software calculates perfusion images, time intensity curve (TIC) analyses and numeric values automatically. The results are exported as Excel data sheet.

For statistical analysis IBM SPSS Statistics for Windows Version 25.0 (Armonk, NY: IBM Corp) was used. Arithmetic mean and standard deviation were calculated for all samples. One factorial ANOVA was used for the 3-sample comparisons with Tukey test for post hoc analyses. p-values lower than 0.05 were considered significant.

## Results

The lesion diameter ranged between 7 and 15 mm (mean 11 ± 3 mm). CEUS revealed 5 hypoechoic irregular lesions as scars, since there was no contrast media uptake. Six lesions were benign. Of these benign lesions one turned out to be a complicated cyst with contrast media uptake of the thickened wall. Four lesions were fibroadenomas with homogenous contrast media enhancement (Fig. 2). One was a necrosis of fatty tissue. One was an atypical ductal hyperplasia (ADH) considered as an intermediate lesion. Twelve lesions were highly suspicious for breast cancer with irregular enhancement at the margins and a partial wash out (Fig. 1a–d). In those 12 cases histopathology showed breast cancer. All the suspicious lesions were correctly identified visually. All lesions (except for the complicated cyst) were biopsied. A follow-up was performed for the cystic lesion after 6 months. There were no changes at all. After tumor surgery there was a follow-up, too. No recurrence was found after 12 months of follow-up.

In all 24 cases CEUS perfusion imaging using external software (VueBox) could detect the changes in micro-vascularization with irregular hyper-vascularization in tumors. Parametric maps in false colors illustrate those differences showing higher enhancement (red/yellow) for the tumor lesions in comparison to the surrounding breast tissue (green/blue). The differences become especially obvious in PE. For malignant lesions there is typically an early and strong contrast agent enhancement with a short TTP < 15 s. The corresponding changes in parametric maps and curve analysis can be demonstrated with an early wash out beginning after 20 s.

A prolonged contrast agent enhancement with lower PE and prolonged wash out is typical for benign lesions. In scars there is typically a reduced enhancement in the center.

PE did not differ between the center of benign lesions (64.2 ± 28.9), malignant lesions (88.1 ± 93.6) and scars (40.0 ± 17.0) (p = 0.428). No differences (p = 0.174) were found for PE values at the margin of benign lesions (96.4 ± 144.9), malignant lesions (54.3 ± 86.2) and scars (203.8 ± 218.9). However, differences (p < 0.001) were found in PE of the surrounding tissue when comparing benign lesions (33.6 ± 25.2) to malignant lesions (15.7 ± 36.3) and scars (277.2 ± 199.9) (Table 1).

**Table 1:** Numeric values for perfusion analysis for Peak Enhancement and AUC for the Regions of Interest (ROI) in the center, at the margin and the surrounding tissue for benign and malignant lesions as well as for scars.

	Peak enhancement			AUC		
	Center	Margin	Surrounding	Center	Margin	Surrounding
Benign	64.2 ± 28.9	96.4 ± 144.9	33.6 ± 25.2*	391.3 ± 213.7	313.3 ± 372.8	151.7 ± 127.8*
Malignant	88.1 ± 93.6	54.3 ± 86.2	15.7 ± 36.3*	314.7 ± 643.9	272.6 ± 566.4	177.9 ± 345.6*
Scar	40.0 ± 17.0	203.8 ± 218.9	277.2 ± 199.9	213.1 ± 124.5	695.0 ± 360.6	1091 ± 693.3
p	0.428	0.174	< 0.001	0.821	0.601	< 0.001

*p < 0.05 versus scar.

There were no significant differences (p = 0.221) in AUC in the center of benign lesions (391.3 ± 213.7), malignant lesions (314.7 ± 643.9) and scars (213.1 ± 124.5). No significant differences (p = 0.601) were found in AUC values of the margin in benign lesions (313.3 ± 372.8), malignant lesions (272.6 ± 566.4) and scars (695.0 ± 360.6). Significant differences (p < 0.01) were found in AUC of the surrounding tissue to benign lesions (151.7 ± 127.8), malignant lesions (177.9 ± 345.6) and scars (1091 ± 1693.3) (Table 1).

There were no differences (p = 0.480) in RT in the center of benign lesions (8.5 ± 4.1), malignant lesions (6.8 ± 6.4) and scars (10.4 ± 5.4). No significant differences (p = 0.337) were found in RT values of the margins of benign lesions (7.4 ± 4.0), malignant lesions (9.4 ± 5.4) and scars (5.5 ± 2.1). No significant differences (p = 0.370) were found for RT values of the surrounding tissue of benign lesions (6.9 ± 4.2), malignant lesions (12.0 ± 12.9) and scars (5.6 ± 2.1) (Table 2).

**Table 2:** Numeric values for perfusion analysis for Rise Time (RT) and mean Transit Time (mTT) and Time to Peak (TTP) for the Regions of Interest (ROI) in the center, at the margin and the surrounding tissue for benign and malignant lesions as well as for scars.

	RT (s)			mTT (s)			TTP (s)		
	Center	Margin	Surrounding	Center	Margin	Surrounding	Center	Margin	Surrounding
Benign	8.5 ± 4.1	7.4 ± 4.0	6.9 ± 4.2	63.7 ± 47.5	84.3 ± 95.1	41.1 ± 31.1	11.6 ± 4.5	9.9 ± 4.6	9.8 ± 5.7
Malignant	6.8 ± 6.4	9.4 ± 5.4	12.0 ± 12.9	111.9 ± 137.3	115.4 ± 93.9	158.3 ± 122.2	9.6 ± 8.0	13.1 ± 8.8	16.9 ± 15.6
Scar	10.4 ± 5.4	5.5 ± 2.1	5.6 ± 2.1	47.2 ± 27.8	39.7 ± 8.1	134.3 ± 193.8	13.7 ± 4.1	9.3 ± 2.1	9.4 ± 1.3
p	0.480	0.337	0.370	0.425	0.327	0.173	0.484	0.521	0.338

Correspondingly, there were no significant differences (p = 0.425) in mTT in the center for benign lesions (63.7 ± 547.5), malignant lesions (111.9 ± 137.3) and scars (47.2 ± 27.8). No significant differences (p = 0.327) were found in mTT values of the margin of benign lesions (84.3 ± 95.1), malignant lesions (115.4 ± 93.9) and scars (39.7 ± 8.1). No significant differences (p = 0.173) were found in mTT values of the surrounding tissue when comparing benign lesions (41.1 ± 31.1) with malignant lesions (158.3 ± 122.2) and scars (134.3 ± 193.8) (Table 2).

Furthermore, there were no significant differences (p = 0.484) in TTP in the center of benign lesions (11.6 ± 4.5), malignant lesions (9.6 ± 8.0) and scars (13.7 ± 4.1). No significant differences (p = 0.521) were found for TTP of the margin of benign lesions (9.9 ± 4.6), malignant lesions (13.1 ± 8.8) and scars (9.3 ± 2.1). No significant differences (p = 0.338) were found in TTP for the surrounding tissue of benign lesions (9.8 ± 5.7), malignant lesions (16.9 ± 15.6) and scars (9.4 ± 1.3) (Table 2).

## Discussion

High-resolution ultrasound is an important diagnostic tool in the interdisciplinary tumor diagnostics. The strength of ultrasound is clearly the detection of focal breast lesions in dense breast tissue (ACR c/d, fibro glandular tissue > 50%) and the safety of the diagnostic tool in younger patients. Furthermore, in a high-risk setting like in hereditary breast cancer, as seen for example in BRCA mutations, lesions can be assessed without radiation exposure. CEUS was performed additionally if there were lesions that could not clearly be classified using B-Mode (and in those preselected cases using ceMRI) and needed further assessment. In B-Mode benign lesions typically do not show any cutaneous changes and grow in a rather horizontal direction. Scars however tend to have cutaneous changes, are hypoechoic and have irregular margins. They do not show typically benign vascularization. As a hint the hypoechoic rim is a typical B-Mode morphology of malignant lesions, however it might be extremely difficult to differentiate a scar from a recurrence. Especially contrast enhanced power Doppler can display tumor vascularization and can hence depict the micro-vascularization. Due to angiogenesis vascularization of malignant lesions is more prominent than in benign masses^14,15^. Sometimes disordered and distorted vessels^16^ can be seen. The caliper of those varies from normal capillaries. This fact can also be used to differentiate between recurrence and post-operative scar, since recurrences show a greater number of vessels and a stronger enhancement. Sometimes penetrating or central vessels can be seen, whereas in scars there are no signs of vascularity inside or nearby^17^. The results of this present pilot study show that there is a significant difference in PE and AUC in the surrounding tissue whereas there was none within the lesions themselves. This might be due to the peritumoral micro-vascularization in the surrounding tissue. As mentioned above there is already an irregular capillarization that can be even displayed with cePower Doppler. Due to the micro-shunts an early wash-out is visible. Malignant lesions tend to have necrosis in the center of the lesions at an early stage. The microbubbles remain strictly intravascular, whereas MR (and CT) contrast media does not remain strictly intravascular but also distributes in the parenchyma. If the tumor micro-vascularization should be observed, a tumor-specific contrast-media is advisable^13^.

The problem with contrast media of the first generation, was that those microbubbles were not stable enough, when using high frequencies that are needed for high resolution breast imaging. The second-generation microbubbles are more stable and are not destroyed as easily as before. However, compared to liver imaging, higher doses of sulfur hexafluoride contrast media (up to 5 ml) are needed for breast imaging, because the higher frequencies still destroy some of the bubbles. The tumor specific contrast media BR-55 is not yet approved for clinical use^13^.

Imaging with CEUS using contrast harmonic imaging (CHI) and low mechanical index (MI < 0.2) is an active field of research and there are promising results in recent studies^18^ (Table 3). CEUS has a diagnostic accuracy of characterizing tumors of up to 90%. Several recent studies suggest that there is a good match between ceMRI and CEUS when rating breast lesions. Though to the present state, characterizing breast tumors using CEUS is not yet recommended in the EFSUMB guidelines for daily clinical use^5^.

**Table 3:** References and literature overview.

Author, Journal, Year, Title	No. of lesions	Conclusion	Accuracy, sensitivity, specificity
Du YR et al., Clinical Hemorheol Microcirculation, 2018 Application of contrast-enhanced ultrasound in the diagnosis of small breast lesions^19^	105	CEUS was useful to differentiate benign from malignant breast lesions	Acc: 80.9 Sens: 78.7 Spec: 84.1
Noro A et al., J Med Ultrasonics 2016 Impact of parametric imaging on contrast-enhanced ultrasound of breast cancer^20^	65	The use of parametric imaging improves visibility of breast cancer	NA
Xiao X et al., PLoS One 2014 Breast contrast-enhanced ultrasound: is a scoring system feasible^21^	839	The contrast-enhancement patterns of benign and malignant breast lesions is different	Acc: 90.8 Sens: 93.7 Spec: 88.7
Luo J et al., World J Radiol 2016 Contrast-enhanced ultrasound improves performance of breast imaging reporting and data system of critical breast lesions^22^	235	The evaluation of BI-RADS 4 lesions with CEUS result in reduced biopsy rates and increased cancer-to-biopsy yields	Sens: 85.4 Spec: 87.8
Luo J et al., World J Radiol 2016 Predictive model for contrast-enhanced ultrasound of the breast^23^	235	The breast CEUS model can predict risk of malignant breast lesions more accurately	Sens: 84.4 Spec: 82.7
Sarocco A et al., Acta radiologica 2012 Differentiation between benign and malignant breast tumor using kinetic features of real-time harmonic contrast-enhanced ultrasound^24^	96	Real-time CEUS can evolve into a new non-invasive option for differentiation of malignant from benign breast lesions	NA
Zhao H et al., European Journal of Radiology 2008 Contrast-enhanced ultrasound is helpful in the differentiation of malignant and benign breast lesions^25^	76	CEUS cooperation with conventional US shows improved accuracy in differentiating between benign and malignant breast tumors	Acc: 90.8 Sens: 86.7 Spec: 96.8
Lee SC et al. J Ultrasound Med 2018 Contrast-enhanced ultrasound imaging of breast masses^26^	131	CEUS may be a valuable modality that can be used to predict benign pathologic results of breast masses	NA
Li C et al., Journal of Biomedical Research 2018 Diagnostic performance of contrast-enhanced ultrasound and enhanced magnetic resonance for breast nodules^16^	120	The combined use of conventional US and CEUS displays a good agreement with MRI in differentiation benign from malignant breast lesions	Acc: 92.5 Sens: 90.1 Spec: 95.9

Above all, it is important to keep in mind, that CEUS can only visualize lesions also seen on conventional ultrasound and one should focus on one lesion at a time when assessing breast lesions. It is impossible to sufficiently scan both sides with just one bolus of contrast-media. CEUS is especially helpful if there is a contraindication towards MRI contrast media, like kidney insufficiency.

It has already been shown, that the classification of breast lesions in regard to malignancy is feasible and comparable to the specificity of MRI, using high resolution ultrasound. The processing time using external perfusion software was 30 min (20–40 min). The color-coded perfusion parameters PE and AUC were the most useful parameters to display a fast contrast media enhancement in suspicious lesions. Those parameters can also be used for follow up after and during neoadjuvant chemotherapy. However, the visual assessment of lesions was sufficient in all the cases of the study. Post-processing with external perfusion software did not add extra value to the examination.

Perfusion imaging can display the dynamics of CEUS in pseudo colors. Areas with fast and high contrast enhancement are shown in red and yellow. Hypo-perfused areas are coded in blue or green. Therefore, it is obvious why CEUS perfusion in the setting of neoadjuvant chemotherapy (NAC) is feasible and comparable to PET-CT. In patients with NAC a decrease in enhancement should be achieved. Using TIC analyses, a (significant) decrease in wash-in/wash-out AUC^26^ as well as an increase in RT^27^ can be seen. Some TIC parameters obtained by CEUS may allow prediction of the response of breast cancer to NAC at a very early point of time^10,28,29^.

Another valuable diagnostic aspect of CEUS is the assessment of the sentinel lymph node in case of metastases of the axillary lymph nodes. Non-metastatic lymph nodes show a homogeneous enhancement pattern^25^, whereas metastatic lymph nodes display an (irregular) enhancement that even goes to hypoperfusion or non-perfusion in some areas^30^. However, the latest guidelines have not yet recommended this method for clinical use^31^.

CEUS with or without additional perfusion analysis is usually accepted for the diagnostics of lesions in solid abdominal organs, especially the liver^32,33^. CEUS adds excellent value to mammography and sonography. However, in breast imaging CEUS will not replace conventional screening mammography and sonography in the next years.

We did not compare the VueBox analyses to standard machine TIC analysis, because even if the same type of machine (LOGIQE9) was used, there are still some differences in the software, that might influence the results of TIC analyses. VueBox has an integrated correction for the transducer and the ultrasound machine and thus makes the results more comparable.

The main limitation of this first pilot study is the small number of cases. Using CEUS with an additional external CEUS perfusion software is not clinical routine and is only indicated for suspicious small non-cystic most likely malignant lesions. High resolution ultrasound technology is necessary, as well as multifrequency linear transducers for CEUS. Digital DICOM loops need to be continuously stored for up to 1 min. Independent reading can be performed using external software but only by experienced readers. This is time consuming and needs up to 60 min per case. Moving artifacts should be reduced during the CEUS examinations itself.

The cost for 2.4 ml of ultrasound contrast agent is up to 60 Euro and additional costs arise for the external software. So far, sulfur hexafluoride microbubbles (SonoVue/Lumason) are used for the majority of CEUS indications in the clinical setting^5,34^. Tumor specific contrast media is in trials. Unfortunately, so far only a region of interest can be defined even though cross beam technology allows for partial 3D imaging. However, so far this external perfusion software cannot calculate volume based perfusion imaging on basis of a real time 3D/4D probe.

High end ultrasound machines often have integrated perfusion software with TIC analysis, for evaluation of PE and AUC for faster dynamic evaluation of tumor micro-vascularization. VueBox is an external perfusion software that can be used with any transducer and any high end ultrasound machine. There is always the correction for the transducer and the machine when performing perfusion analysis with VueBox. Consequently, this pilot study aimed to demonstrate the potential of external perfusion software (VueBox) for independent reading of DICOM loops in solid non-cystic breast lesions. The integrated perfusion software analysis tools can only be used with one machine.

To sum it up, CEUS with BI-RADS can assess the risk of malignant lesions more accurately and can therefore downgrade BI-RADS 4 lesions^26^. There is no additional diagnostic value for the primary diagnostics when using additional perfusion software. Further research is needed to evaluate the importance of CEUS in breast imaging in daily clinical use. The relevant CEUS perfusion parameters of breast tumor micro-vascularization like PE and AUC can usually be evaluated by high end machines with integrated perfusion software. Other perfusion parameters evaluated by an external software might be taken in consideration for the evaluation of therapeutic effects.

## References

[1] Sprague BL (2015). Benefits, harms, and cost-effectiveness of supplemental ultrasonography screening for women with dense breasts. Ann. Intern. Med..

[2] Hooley RJ (2012). Screening US in patients with mammographically dense breasts: Initial experience with Connecticut Public Act 09–41. Radiology.

[3] Carney PA (2003). Individual and combined effects of age, breast density, and hormone replacement therapy use on the accuracy of screening mammography. Ann. Intern. Med..

[4] Mann RM (2015). Breast MRI: EUSOBI recommendations for women's information. Eur. Radiol..

[5] Sidhu PS (2018). The EFSUMB Guidelines and Recommendations for the Clinical Practice of Contrast-Enhanced Ultrasound (CEUS) in non-hepatic applications: Update 2017 (Short Version). Ultraschall Med..

[6] Balleyguier C (2009). New potential and applications of contrast-enhanced ultrasound of the breast: Own investigations and review of the literature. Eur. J. Radiol..

[7] Quan J (2019). The clinical role of contrast enhanced ultrasound in differential diagnosis of BI-RADS 4 breast disease. Clin. Hemorheol. Microcirc..

[8] Shao SH (2020). Incorporation of contrast-enhanced ultrasound in the differential diagnosis for breast lesions with inconsistent results on mammography and conventional ultrasound. Clin. Hemorheol. Microcirc..

[9] Dobruch-Sobczak K (2021). Multiparametric ultrasound examination for response assessment in breast cancer patients undergoing neoadjuvant therapy. Sci. Rep..

[10] Wan CF (2018). Quantitative contrast-enhanced ultrasound evaluation of pathological complete response in patients with locally advanced breast cancer receiving neoadjuvant chemotherapy. Eur. J. Radiol..

[11] Liu J (2019). Percutaneous contrast-enhanced ultrasound for localization and diagnosis of sentinel lymph node in early breast cancer. Sci. Rep..

[12] Lamby P (2017). Effect of iodinated contrast media on renal perfusion: A randomized comparison study in pigs using quantitative contrast-enhanced ultrasound (CEUS). Sci. Rep..

[13] Bitterer F (2020). In vivo detection of breast cancer liver metastases in humanized tumour mice using tumour specific contrast agent BR55®. Clin. Hemorheol. Microcirc..

[14] Engels K, Fox SB, Whitehouse RM, Gatter KC, Harris AL (1997). Distinct angiogenic patterns are associated with high-grade in situ ductal carcinomas of the breast. J. Pathol..

[15] Madjar H, Prömpeler HJ, Sauerbrei W, Wolfarth R, Pfleiderer A (1994). Color Doppler flow criteria of breast lesions. Ultrasound. Med. Biol..

[16] Li CY (2018). Diagnostic performance of contrast-enhanced ultrasound and enhanced magnetic resonance for breast nodules. J. Biomed. Res..

[17] Aichinger U, Schulz-Wendtland R, Krämer S, Lell M, Bautz W (2002). Scar or recurrence–comparison of MRI and color-coded ultrasound with echo signal amplifiers. RoFo Fortschritte auf dem Gebiete der Rontgenstrahlen und der Nuklearmedizin.

[18] Hu W (2020). The clinical value of Arrival-time Parametric Imaging using contrast-enhanced ultrasonography in differentiating benign and malignant breast lesions. Clin. Hemorheol. Microcirc..

[19] Du YR, Wu Y, Chen M, Gu XG (2018). Application of contrast-enhanced ultrasound in the diagnosis of small breast lesions. Clin. Hemorheol. Microcirc..

[20] Noro A (2016). Impact of parametric imaging on contrast-enhanced ultrasound of breast cancer. J. Med. Ultrason..

[21] Xiao X, Ou B, Yang H, Wu H, Luo B (2014). Breast contrast-enhanced ultrasound: Is a scoring system feasible? A preliminary study in China. PLoS One.

[22] Luo J (2016). Contrast-enhanced ultrasound improved performance of breast imaging reporting and data system evaluation of critical breast lesions. World J. Radiol..

[23] Luo J (2016). Predictive model for contrast-enhanced ultrasound of the breast: Is it feasible in malignant risk assessment of breast imaging reporting and data system 4 lesions?. World J. Radiol..

[24] Saracco A (2012). Differentiation between benign and malignant breast tumors using kinetic features of real-time harmonic contrast-enhanced ultrasound. Acta Radiol..

[25] Zhao H (2010). Contrast-enhanced ultrasound is helpful in the differentiation of malignant and benign breast lesions. Eur. J. Radiol..

[26] Lee SC (2019). Contrast-enhanced ultrasound imaging of breast masses: Adjunct tool to decrease the number of false-positive biopsy results. J. Ultrasound Med..

[27] Kim Y (2018). Early prediction of response to neoadjuvant chemotherapy using dynamic contrast-enhanced MRI and ultrasound in breast cancer. Korean J. Radiol..

[28] Lee YJ, Kim SH, Kang BJ, Kim YJ (2019). Contrast-enhanced ultrasound for early prediction of response of breast cancer to neoadjuvant chemotherapy. Ultraschall. Med..

[29] Jia WR (2016). Three-dimensional contrast-enhanced ultrasound in response assessment for breast cancer: A comparison with dynamic contrast-enhanced magnetic resonance imaging and pathology. Sci. Rep..

[30] Xie F (2015). Intradermal microbubbles and contrast-enhanced ultrasound (CEUS) is a feasible approach for sentinel lymph node identification in early-stage breast cancer. World J. Surg. Oncol..

[31] Săftoiu A (2019). The EFSUMB guidelines and recommendations for the clinical practice of elastography in non-hepatic applications: Update 2018. Ultraschall. Med..

[32] Dietrich CF (2020). Guidelines and good clinical practice recommendations for contrast-enhanced ultrasound (CEUS) in the liver-update 2020 WFUMB in cooperation with EFSUMB, AFSUMB, AIUM, and FLAUS. Ultrasound Med. Biol..

[33] Wildner D (2019). Differentiation of malignant liver tumors by software-based perfusion quantification with dynamic contrast-enhanced ultrasound (DCEUS). Clin. Hemorheol. Microcirc..

[34] Sidhu PS (2018). The EFSUMB guidelines and recommendations for the clinical practice of contrast-enhanced ultrasound (CEUS) in non-hepatic applications: Update 2017 (Long Version). Ultraschall. Med..

